# YAP/TAZ interacts with RBM39 to confer resistance against indisulam

**DOI:** 10.1038/s41389-024-00527-0

**Published:** 2024-07-15

**Authors:** Toshinori Ando, Kento Okamoto, Yume Ueda, Nanako Kataoka, Tomoaki Shintani, Souichi Yanamoto, Mutsumi Miyauchi, Mikihito Kajiya

**Affiliations:** 1 Center of Oral Clinical Examination, Hiroshima University Hospital, Hiroshima, 734-8551 Japan; 2 Department of Oral Oncology, Graduate School of Biomedical and Health Sciences, Hiroshima University, Hiroshima, 734-8553 Japan; 3 Department of Oral and Maxillofacial Pathobiology, Graduate School of Biomedical and Health Sciences, Hiroshima University, Hiroshima, 734-8553 Japan

**Keywords:** HIPPO signalling, Head and neck cancer

## Abstract

The Hippo pathway and its downstream effectors, Yes-associated protein/transcriptional coactivator with PDZ-binding motif (YAP/TAZ), are essential for cell growth and organ development. Emerging evidence revealed that the Hippo pathway and YAP/TAZ are frequently dysregulated by multiple genetic alterations in solid cancers including head and neck squamous cell carcinoma (HNSCC); however, the YAP/TAZ-nuclear interactome remains unclear. RNA-binding motif protein 39 (RBM39) enhances transcriptional activity of several transcription factors and also regulates mRNA splicing. Indisulam degrading RBM39 induces alternative splicing, leading to cell death. However, clinical trials of indisulam have failed to show effectiveness. Therefore, clarifying the resistance mechanism against splicing inhibitors is urgently required. In this study, we identified RBM39 as a novel YAP/TAZ-interacting molecule by proteome analysis. RBM39 promoted YAP/TAZ transcriptional activity. We further elucidated that indisulam reduces RBM39/YAP/TAZ-mediated integrin or collagen expression, thereby inactivating focal adhesion kinase (FAK) important for cell survival. Moreover, indisulam also induced alternative splicing of cell cycle- or DNA metabolism-related genes. YAP/TAZ hyperactivation delayed indisulam-induced RBM39 degradation, which restored the integrin/collagen expression to activate FAK, and alternative splicing, thereby conferring resistance against indisulam in vitro and in vivo. Our findings may aid to develop a novel cancer therapy focusing on YAP/TAZ/RBM39 interaction.

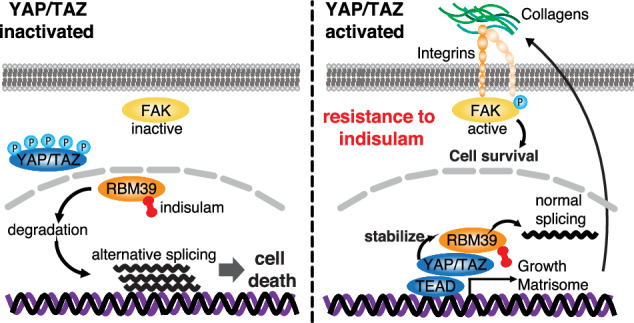

## Introduction

The Hippo pathway and its downstream effectors, yes-associated protein and transcriptional co-activator with PDZ binding motif (YAP/TAZ) play a central role in cell growth and organ development [1]. When the hippo pathway is active, LATS1/2, the core kinases of the Hippo pathway, phosphorylates YAP/TAZ leading to cytoplasmic retention or degradation. When the hippo pathway is inactive, dephosphorylated YAP/TAZ localizes into the nucleus, binds to TEA domain family members (TEAD), promoting transcription of growth-related genes, including *connective tissue growth factor* (*CTGF*) and *cysteine-rich angiogenic inducer 61* (*CYR61*) [2]. Genetic alterations dysregulate the Hippo pathway followed by aberrant YAP/TAZ activation, which is prevalent in solid cancers including head and neck squamous cell carcinoma (HNSCC). For example, HNSCC harbors *YAP1* or *EGFR* amplification, and *FAT1* loss/mutation, resulting in YAP/TAZ activation [3, 4]. Moreover, emerging evidence suggest that YAP/TAZ interacts with several nuclear factors including E2F, activator protein-1 (AP-1) and bromodomain containing protein 4 to synergistically promote cell proliferation [5–7]. However, the YAP/TAZ-nuclear interactome is not fully understood.

RNA-binding motif protein 39 (RBM39) interacts with transcription factors including c-Jun, a component of AP-1, estrogen receptor α, progesterone receptor, and nuclear receptor coactivator 6, enhancing their transcriptional activities [8]. RBM39 also functions as components of the spliceosome regulating pre-mRNA splicing [8]. Indisulam and E7820, aryl sulfonamides, induce RBM39 degradation by DCAF15 recruitment, thereby inducing alternative splicing that leads to cell death [9, 10]. Since alternative splicing increases neoantigens in cancer cells, indisulam can be combined with immune checkpoint inhibitors (ICIs) [11]. However, a single treatment with indisulam had unexpectedly shown less effectiveness in patients with solid cancers including head and neck squamous cell carcinoma (HNSCC), melanoma, and non-small cell lung cancer in phase I or II clinical trials [8]. Thus, there is an urgent need to clarify the resistance mechanism before the indisulam can be used clinically.

In this study, we identified RBM39 as a novel YAP/TAZ interactor in the nucleus. Notably, we demonstrated that YAP/TAZ interact with RBM39 to confer resistance to indisulam. Our findings decipher the resistance mechanism of indisulam, which may aid the development of a novel therapeutic approach for patients with solid cancers including HNSCC.

## Results

### RBM39 promotes YAP/TAZ transcriptional activity and YAP/TAZ active cells are resistant to indisulam

**Fig. 1:**
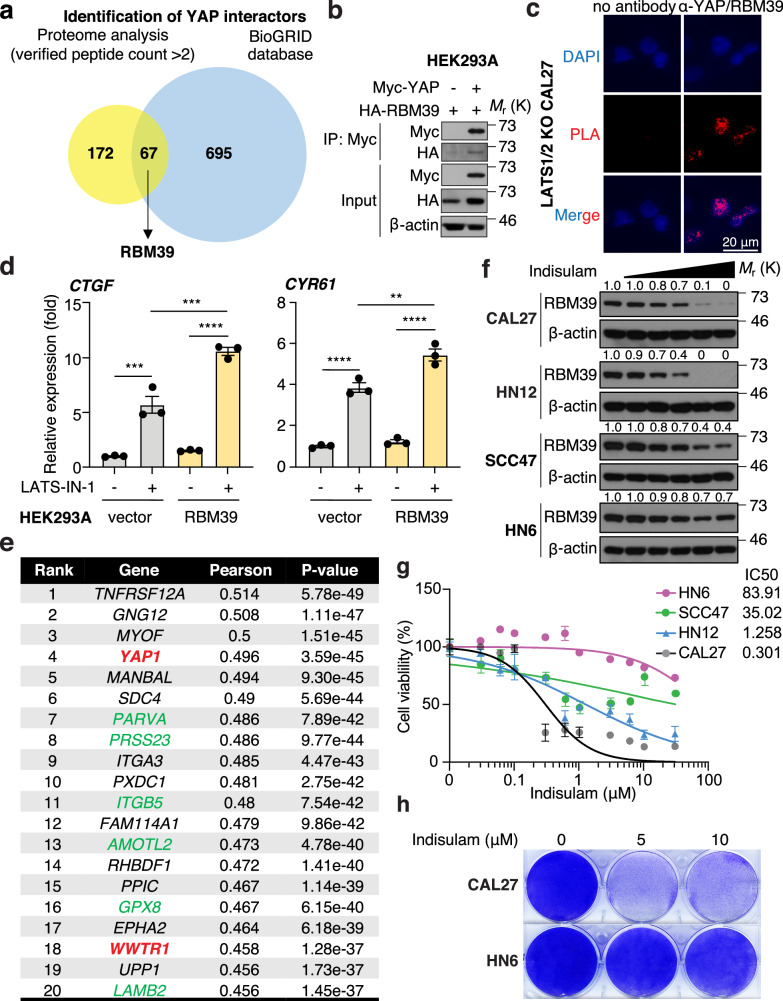
RBM39 promotes YAP/TAZ transcriptional activity and YAP/TAZ active cells are resistant to indisulam. **a** Schematic representation of RBM39 identification. Lysate was co-immunoprecipitated with anti-YAP antibody, then analyzed by proteome analysis. The result was compared with the BioGRID database. **b** Western blot of HEK293A cells overexpressing Myc-YAP and HA-RBM39. Lysate was co-immunoprecipitated with anti-Myc antibody. **c** Immunofluorescence of proximity ligation assay in LATS1/2 KO CAL27 cells. The red signal suggests the protein interaction. The antibodies against YAP and RBM39 were used. **d** Relative mRNA expression in HEK293A cells overexpressing vector or RBM39. Cells were transfected with the vectors, then treated with LATS-IN-1 (10 μM) for 24 h. (*N* = 3). **e** The DEPMAP shows the top 20 gene expression conferring resistance to indisulam (Pearson ratio is gene expression vs drug sensitivity area under the curve (AUC)). *YAP1* and *WWTR1* are highlighted in red, and YAP/TAZ-targeted genes in green. **f** Western blot of HNSCC cell lines. Cells were treated with indisulam at 0, 0.01, 0.05, 0.1, 0.5, and 1 μM for 24 h. **g** Cell viability of HNSCC cell lines. (*N* = 5). **h** Crystal violet staining of viable HNSCC cells. ANOVA with Tukey–Kramer post hoc test was used. Mean ± SEM (**d**); ****, *P* < 0.0001; ***, *P* < 0.001; **, *P* < 0.01. Protein level of RBM39 was compared to without indisulam treatment (**f**).

We previously generated LATS1/2 knockout (KO) CAL27 cells, a human HNSCC cell line [4]. We performed proteome analysis of nuclear molecules bound to YAP using LATS1/2 KO CAL27 cells showing constitutively active YAP located in the nucleus. We compared the results of proteomic analysis and YAP-binding molecules in the BioGRID database, and identified 67 proteins. Then, we focused on RBM39 as a not yet validated YAP-interacting molecule (Fig. 1a and Supplementary Table 1). We used a co-immunoprecipitation assay to verify the binding between YAP and RBM39 (Fig. 1b). YAP and RBM39 co-localized in the nucleus in LATS1/2 KO CAL27 cells (Supplementary Fig. 1a). In addition, proximity ligation assay demonstrated that YAP and RBM39 interact in the nucleus (Fig. 1c). Because RBM39 is known to enhance transcriptional activity of several transcription factors, we examined whether RBM39 promotes YAP/TAZ/TEAD transcriptional activity. LATS-IN-1, an inhibitor of LATS1/2, treatment increased *CTGF* and *CYR61* expression in vector-expressing HEK293A cells, whereas LATS-IN-1 further increased *CTGF*/*CYR61* expression in RBM39-overexpressing cells (Fig. 1d). This is due to the stabilization of YAP/TAZ proteins by overexpressed RBM39 (Supplementary Fig. 1b). However, RBM39 overexpression alone failed to increase basal expression level of *CTGF*/*CYR61* because YAP/TAZ are inactivated in serum-starved condition, suggesting that RBM39 alone does not have function to promote transcription of *CTGF*/*CYR61* (Fig. 1d). These results suggest that RBM39 promotes YAP/TAZ/TEAD transcriptional activity.

Next, we speculated that the YAP/TAZ/RBM39 interaction might alter indisulam sensitivity. The cancer dependency map (DEPMAP) showed that *YAP1* and *WWTR1* genes, encoding YAP and TAZ, respectively, were among the top 20 genes related to indisulam resistance (Fig. 1e and Supplementary Fig. 1c). As previously reported, *DCAF15* was among the top 20 genes related to indisulam sensitivity, and *RBM39* showed no correlation with indisulam sensitivity (Supplementary Fig. 1c, d). We examined the YAP/TAZ activity and indisulam sensitivity in HNSCC cell lines. CAL27 cells showed the lowest *CTGF*/*CYR61* expression and representative YAP/TAZ-targeted genes, whereas HN6 and SCC47 cells showed the highest expression. HN12 cells showed high *CTGF* expression but low *CYR61* expression (Supplementary Fig. 1e). RBM39 protein levels and *DCAF15* mRNA expression were not significantly different among HNSCC cell lines (Supplementary Fig. 1e, f). Several reports have also shown that neuroblastoma, leukemia, myeloma, and lymphoma are sensitive to indisulam due to their high expression of *DCAF15* [10, 12, 13]. However, *YAP1* expression is extremely low in these cancer types, suggesting that cancer cells harboring low YAP activity may be sensitive to indisulam (Supplementary Fig. 2a, b). Indisulam readily reduced RBM39 protein levels in CAL27 and HN12 in a dose-dependent manner. In contrast, the protein levels in HN6 and SCC47 cells were sustained, although the levels are decreased (Fig. 1f). CAL27 and HN12 cells were sensitive to indisulam, whereas HN6 and SCC47 cells were resistant to indisulam (Fig. 1g, h). Collectively, YAP/TAZ active cells are resistant to splicing inhibitors due to their high stability of RBM39.

### YAP/TAZ delay RBM39 degradation and promote integrin, collagen, and FAK activation to be resistant to indisulam

**Fig. 2:**
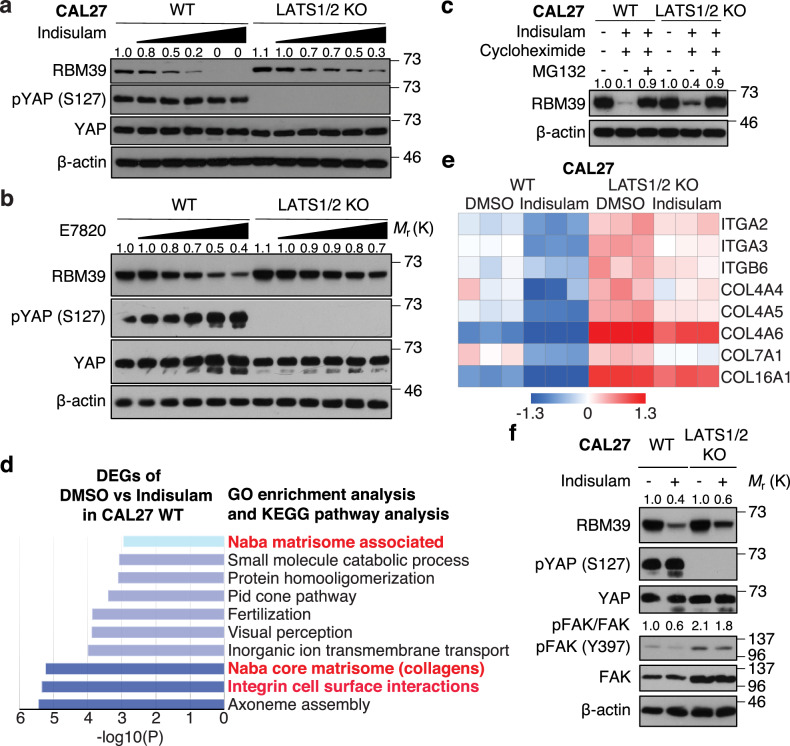
YAP/TAZ delay RBM39 degradation and promote integrin, collagen, and FAK activation to be resistant to indisulam. **a** Western blot of WT and LATS1/2 KO CAL27 cells treated with indisulam at 0, 0.01, 0.05, 0.1, 0.5, and 1 μM for 24 h. **b** Western blot treated with E7820. **c** Western blot of WT and LATS1/2 KO CAL27 cells treated with indisulam at 1 μM, cycloheximide at 200 μg/mL, and MG132 at 10 μM for 8 h. **d** GO and KEGG pathway enrichment analysis of DEGs in WT CAL27 cells treated with DMSO or indisulam. **e** Heatmap of integrins and collagens expression. **f** Western blot of WT and LATS1/2 KO CAL27 cells. Cells were treated with indisulam at 1 μM for 24 h. Protein level of RBM39 was compared to WT CAL27 cells without indisulam, E7820, cycloheximide, or MG132 treatment (**a**–**c**, **f**).

We attempted to clarify the mechanism by which YAP/TAZ activation confers resistance to indisulam. RBM39 protein levels were rapidly decreased by indisulam or E7820 in wild-type (WT) CAL27 and HN12 cells but were sustained at higher level in LATS1/2 KO CAL27 and HN12 cells, although the levels were reduced (Fig. 2a, b, and Supplementary Fig. 2c). Compared to indisulam, E7820 induced faster degradation of RBM39 (Supplementary Fig. 2d). In comparison with WT CAL27 cells, RBM39 degradation by indisulam under cycloheximide treatment was delayed in LATS1/2 (Fig. 2c). To comprehensively examine transcriptional changes by indisulam in HNSCC cells, we performed RNA-seq of WT and LATS1/2 KO CAL27 cells with DMSO or indisulam treatment. Gene Ontology and KEGG pathway enrichment analysis revealed that differentially expressed genes (DEGs) in DMSO vs indisulam were enriched into “integrin cell surface interactions” or “Naba core matrisome including collagens” (Fig. 2d). We then focused on integrins and collagens followed by focal adhesion kinase (FAK) activation important for cell survival and proliferation [14], which showed resistance to indisulam in DEPMAP (Supplementary Fig. 2e). Indisulam suppressed the expression of integrins: *ITGA2*, *ITGA3*, and *ITGB6*, and collagens *COL4A4*, *COL4A5*, *COL4A6*, *COL7A1*, *COL16A1* in WT CAL27 cells. In contrast, these integrin and collagen expression were sustained at high levels in LATS1/2 KO CAL27 cells, although the levels were reduced (Fig. 2e). Moreover, indisulam reduced FAK phosphorylation (inactivated) in WT CAL27 cells, whereas FAK remained highly phosphorylated (activated) in LATS1/2 KO cells (Fig. 2f). *PTK2*, encoding FAK, was also positively correlated with indisulam resistance in DEPMAP (Supplementary Fig. 2e). In short, indisulam reduce RBM39/YAP/TAZ-induced integrins/collagens expression converging into FAK activation, which can be restored by YAP/TAZ hyperactivation.

### LATS1/2 KO-driven YAP/TAZ activation suppresses indisulam-induced alternative splicing

**Fig. 3:**
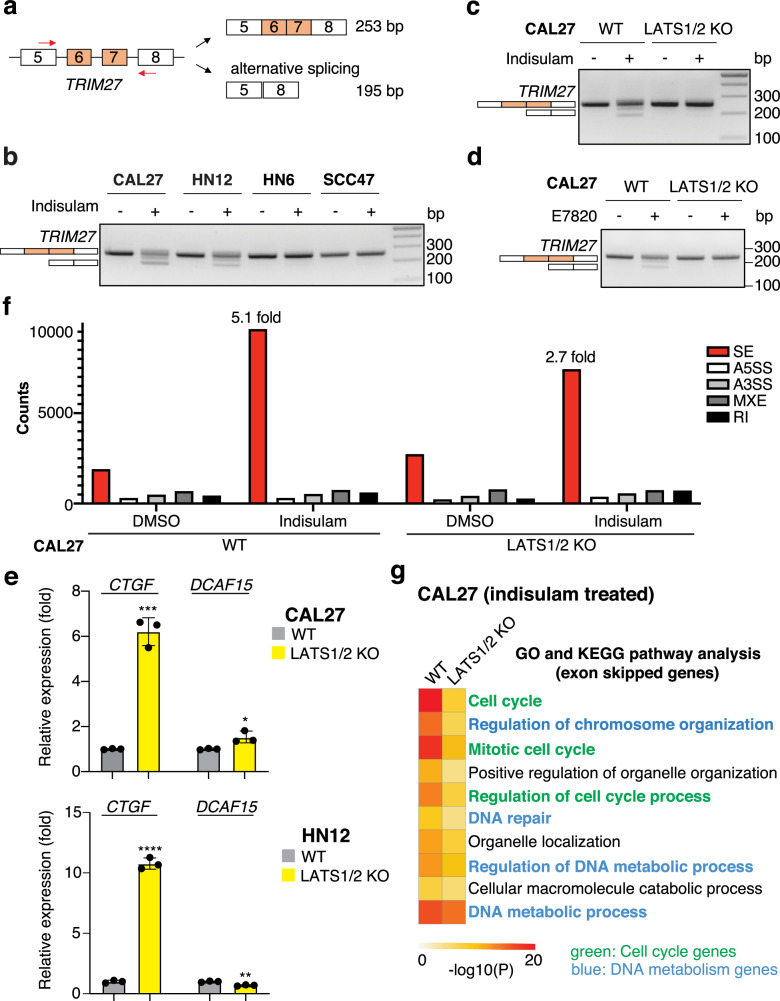
LATS1/2 KO-driven YAP/TAZ activation suppresses indisulam-induced alternative splicing. **a** Schematic representation of the PCR products of *TRIM27* detecting alternative splicing. **b** Alternative splicing of *TRIM27* in HNSCC cell lines. **c** Alternative splicing of *TRIM27* in WT and LATS1/2 KO CAL27 cells treated with indisulam. (**d**) Alternative splicing treated with E7820. (**e**) Relative mRNA expression of *CTGF* and *DCAF15* in WT and LATS1/2 KO CAL27 cells (N = 3). (**f**) Comprehensive splicing analysis of DMSO or indisulam-treated WT and LATS1/2 KO CAL27 cells. SE skipped exon, A5SS alternative 3’ splice site, A3SS alternative 5’ splice site, MXE mutually excluded exon, RI retained intron. (**g**) GO and KEGG pathway enrichment analysis of the SE genes. Cell cycle-related are highlighted in green, and DNA-metabolism related are in blue. Student’s t-test was used. Mean ± SEM (**e**); ****, *P* < 0.0001; ***, *P* < 0.001; **, *P* < 0.01.

Indisulam induces alternative splicing in cancer cells. Since *tripartite motif containing 27* (*TRIM27*) is one of the representative exon-skipped genes induced by indisulam treatment in previous reports, we assessed the indisulam-mediated alternative splicing of *TRIM27* in HNSCC cell lines [10, 12] (Fig. 3a). CAL27 and HN12 cells showed exon skipping of *TRIM27*, whereas HN6 and SCC47 cells showed almost no effect (Fig. 3b). In addition, WT CAL27 and HN12 cells showed alternative splicing of *TRIM27*, whereas LATS1/2 KO CAL27 and HN12 cells did not (Fig. 3c, d, and Supplementary Fig. 3a). To examine the effect of YAP/TAZ activation on *DCAF15* expression, we compared *DCAF15* expression in WT and LATS1/2 KO cells. Although *CTGF* expression was higher in LATS1/2 KO CAL27 and HN12 cells than in WT cells, *DCAF15* expression was not consistent in both cell lines; it was higher in CAL27 and lower in HN12 cells (Fig. 3e). These results suggest that LATS1/2 KO suppresses indisulam-induced RBM39 degradation and alternative splicing, independently of *DCAF15* expression. Furthermore, we performed a comprehensive splicing analysis using the RNA-seq data. Among various types of splicing events, including skipped exon (SE), alternative 5’ splice site (A5SS), alternative 3’ splice site (A3SS), mutually excluded exon (MXE), and retained intron (RI), SE is the most frequent event induced by indisulam. Indisulam dramatically increased SE in WT CAL27 cells (5.1-fold) but only slightly increased SE in LATS1/2 KO (2.7-fold) (Fig. 3f). Gene Ontology and KEGG pathway enrichment analysis of SE in WT CAL27 cells by indisulam revealed that SE is enriched in the cell cycle or DNA metabolism (Fig. 3g). In summary, indisulam induces alternative splicing of cell cycle- or DNA metabolism-related genes, which can be suppressed by YAP/TAZ hyperactivation.

### LATS1/2 KO-driven YAP/TAZ activation confers resistance against indisulam in vitro and in vivo

**Fig. 4:**
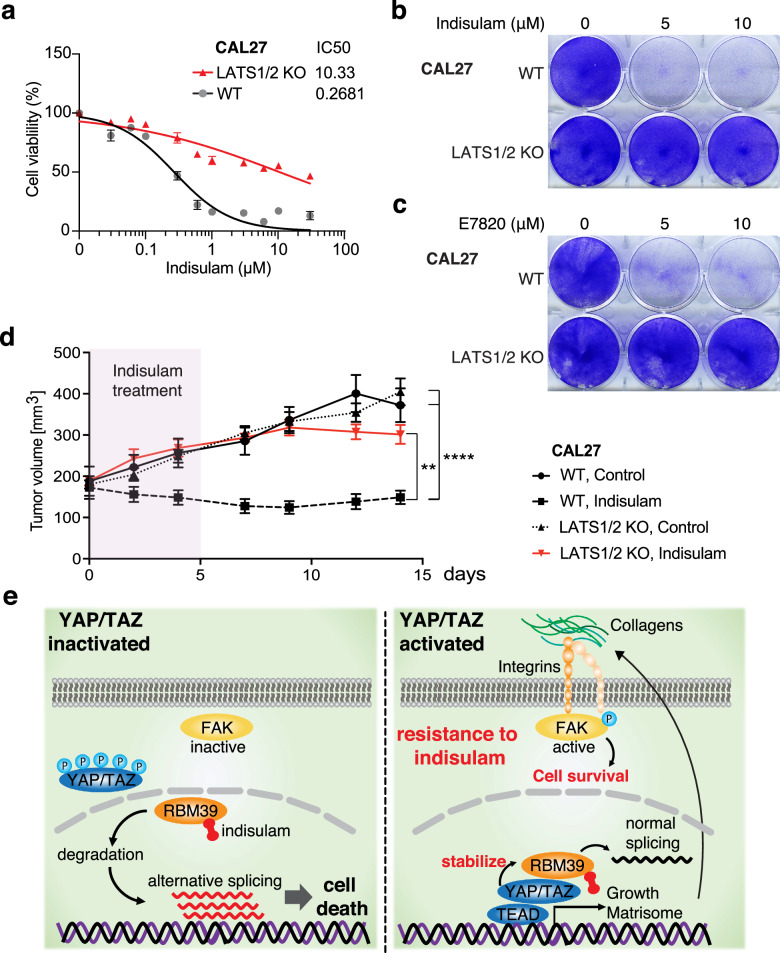
LATS1/2 KO-driven YAP/TAZ activation confers resistance against indisulam in vitro and in vivo. **a** Cell viability of WT and LATS1/2 KO CAL27 cells. (*N* = 3). **b** Crystal violet staining of viable WT and LATS1/2 KO CAL27 cells treated with indisulam. **c** Crystal violet staining treated with E7820. **d** Average growth curves for WT and LATS1/2 KO CAL27 cells transplanted into athymic nude mice. The tumors were treated with indisulam for 6 days (*N* = 10 per group). **e** Summary of the mechanism. ANOVA with Tukey–Kramer post hoc test was used. Mean ± SEM (**d**); ****, *P* < 0.0001; **, *P* < 0.01.

Finally, we examined whether YAP/TAZ confer resistance to indisulam in vitro and in vivo experiments. WT CAL27 and HN12 cells were sensitive to indisulam or E7820 treatment, whereas LATS1/2 KO CAL27 and HN12 cells were resistant (Fig. 4a–c, and Supplementary Fig. 3b, c). LATS1/2 KO-sustained high RBM39 protein level was reduced by YAP siRNA knockdown (Supplementary Fig. 3d). Moreover, YAP knockdown re-sensitized LATS1/2 KO cells against indisulam (Supplementary Fig. 3e). In addition, RBM39 knockdown by siRNA induced alternative splicing and suppressed proliferation, which indicates that the tumor suppressive effect of indisulam depends on RBM39, not by targeting other molecules. (Supplementary Figs. 3f–i). Furthermore, using an in vivo xenograft model, indisulam treatment inhibited the growth of WT CAL27 cells but not of LATS1/2 KO CAL27 cells (Fig. 4d).

## Discussion

We demonstrated that indisulam degrades RBM39 to inactive FAK, and induce alternative splicing of cell cycle- or DNA metabolism-related genes in YAP/TAZ-inactivated HNSCC cells. In contrast, YAP/TAZ activation stabilizes RBM39 even with indisulam treatment, promotes the transcription of integrins and collagens, resulting in FAK activation, and suppresses alternative splicing, which confers resistance to indisulam (Fig. 4e).

Indisulam unexpectedly failed to show marked responses in multiple Phase I and II clinical trials targeting colon cancer, non-small cell lung cancer, HNSCC, or melanoma [8]. However, several studies have demonstrated that cancers derived from hematopoietic and lymphoid lineages, or high-risk neuroblastomas, are sensitive to indisulam because of their high expression levels of DCAF15, by targeting RNA splicing [10, 12, 13]. Our results showed that YAP/TAZ are new key factors conferring resistance to indisulam, independently of DCAF15 in HNSCC cells. Given that YAP/TAZ are highly activated in solid cancers, including HNSCC, indisulam resistance observed in clinical trials may be attributed to YAP/TAZ. In addition, indisulam has attracted attention because they are expected to promote the response to ICIs [11]. However, YAP/TAZ-hyperactivated solid cancers may be resistant to indisulam, indicating that combination therapy with indisulam and ICIs may be ineffective in patients with solid cancers.

Indisulam binds to one of the RNA-recognition motifs (RRMs) of RBM39, RRM2 domain, and a shallow pocket of DCAF15 [15–18]. Given the interaction between other transcription factors and RBM39 [8], YAP/TAZ may also interact with the C-terminal side of RBM39. However, further structural analysis will be required to elucidate how RBM39 interacts with YAP/TAZ to promote their transcriptional activity, and how YAP/TAZ interferes with the complex formation of RBM39, DCAF15, and indisulam, which may yield a new approach that interferes with the interaction between RBM39 and YAP/TAZ to maximize the effect of indisulam, and future combination therapy with ICIs.

## Methods

### Antibodies and reagents

Anti-pYAP (S127) (#4911), anti-YAP (#14074), anti-pFAK (#8556), and FAK (#3285) were purchased from Cell Signaling Technology (MA, USA). β-actin (#A2228) was purchased from Sigma-Aldrich Inc. (MO). Anti-RBM39 (#SC-376531) was purchased from Santa Cruz (MO). Indisulam (#S9742) was purchased from Selleck Chemicals (TX, USA). LATS-IN-1 (#HY-138489) and E7820 (#HY-14571) was purchased from MedChemExpress (NJ). Cycloheximide and MG132 were purchased from FUJIFILM Wako Chemicals (Osaka, JP).

### DNA constructs

Plasmid pRP-CMV-HA-RBM39 encoding human RBM39 was designed and purchased from Vector Builder (JP). GFP-expressing vector was used as control.

### CRISPR/Cas9 genome editing

LATS1/2 KO cells were created using pLentiCRISPRv2 expressing CAS9 and sgRNA described previously [4].

### Proteome analysis

One sample of 500 μg of protein from LATS1/2 KO CAL27 cells was used for co-immunoprecipitation analysis. For immunoprecipitation, cells were lysed in CHAPS buffer (1% CHAPS, 30 mM Tris-HCl, 150 mM NaCl) with protease and phosphatase inhibitor (#78444 Thermo Fisher Scientific, CO) and 1 mM DTT, incubated on ice for 15 min, and centrifuged at 16,000×*g* for 15 min at 4 °C. The supernatants were incubated with anti-YAP antibody (#14074 CST) for 24 h at 4 °C, then incubated with protein A agarose beads for 1 h at 4 °C. Beads were centrifuged and rinsed with CHAPS buffer 5 times. The beads were resuspended in 100 μL of 50 mM Tris-HCl pH8.0 and sent to the company (Promega, Madison, WI, USA). Peptide false discovery rate (FDR) < 1% and protein FDR < 1% are applied for the candidate proteins. The result was compared with BioGRID database (https://thebiogrid.org).

### Cell culture and transfection

CAL27, HN12, and HN6 cells were kindly provided by J Silvio Gutkind, and their identifies were confirmed by STR profiling. They were also verified to be free of mycoplasma infection. CAL27, HN12, and HN6 cells were cultured in Dulbecco’s Modified Eagle Medium (D-6429, Sigma-Aldrich Inc., St. Louis, MO) supplemented with 10% Fetal Bovine Serum (Sigma-Aldrich Inc., St. Louis, MO), 1x antibiotic/antimycotic solution (Sigma-Aldrich Inc., MO) and 5 μg/mL plasmocin^TM^ prophylactic (InvivoGen, CA).

### Western blotting and immunoprecipitation

Western blotting and co-immunoprecipitation were performed as described previously [4]. Densitometry analysis of the bands was performed using “image J software”.

### In situ proximity ligation assay

LATS1/2 KO CAL27 cells were seeded on the cover glass and cultured. After 24 h, cells were fixed in 4% paraformaldehyde in PBS for 10 min at room temperature. Cells were permeabilized using 0.5% Triton X-100 with 200 mM glycine for 10 min at room temperature. In situ PLA was performed with DuoLink In Situ Reagents (Sigma-Aldrich Inc, MO) according to the manufacturer’s instructions. We used the primary antibody against YAP and RBM39.

### Cell viability assay

The cells were seeded in 96 well plates. The attached cells were treated with DMSO or indisulam for 3 days. CellTiter-Blue Cell Viability Assay reagent (Promega, Madison, WI, USA) was applied. The cells were then incubated with reagent for 4 h. The absorbance was measured using a microplate reader.

### Crystal violet staining

The cells were seeded in 6 well plates. The attached cells were fixed with 4% formalin for 20 min, then stained with 0.5% crystal violet solution containing 20% methanol for 5 min.

### RNA isolation and alternative splicing

RNA extraction was performed using the RNeasy Mini Kit following the manufacturer’s instructions (#74104, Qiagen, Hilden, Germany). The SuperScript^TM^ VILO^TM^ cDNA Synthesis Kit (#11754250, Thermo Fisher Scientific, CO, USA) was used for cDNA synthesis. The following primers were used for alternative splicing. *TRIM27* F: 5’ GCAGGTCCTGTTGGAGGTAA 3’, R: CCTGAACCTTGGATCACACC 3’ Total cDNA was amplified using Fast SYBR Green Master Mix (#4385612, Thermo Fisher Scientific, CO, USA).

### Real-time PCR

RNA extraction and cDNA synthesis was performed as explained above. Real-time PCR and the primer sequences of CTGF/CYR61 were same as previously described [19]. The following primers were used for *DCAF15*. F: 5’ CCAGCCTGGCTATGTCAACT 3’, R: 5’ TCTTGTCGTCCTCCAACTCAT 3’.

### RNA-seq

The cells were treated with DMSO or indisulam at 1 μM for 24 h (N = 3). RNA-seq samples were sequenced using the Illumina platform. For each sample, paired-end sequencing reads were mapped using HISAT2 version 2.1.0 to the hg38 reference human genome downloaded from Ensembl. To compute transcript abundance, uniquely mapped reads were quantified using featureCounts version 1.6.3. Differential gene expression analysis was performed using DESeq2 version 1.38.3, using a parametric fit. Metascape version 3.5.20230101 was used for Gene Ontology and KEGG pathway enrichment analysis [20]. MORPHEUS (https://software.broadinstitute.org/morpheus) was used to generate a heat map. The samples were sequenced using the Illumina platform for the splicing analysis. For each sample, paired-end sequencing reads were mapped using STAR version 2.1.0 to the hg38 reference human genome downloaded from Ensembl. RNA-Seq Multivariate Analysis of Transcript Splicing (rMATS) was used to detect alternative splicing events, where exon and intron regions were defined from the hg38 human reference genome. To estimate differential exon-skipping events, the number of exon-junction reads and exon-skipping reads were calculated and compared with DMSO-treated WT CAL27 cells. Alternatively, the numbers of exon-intron and exon-exon reads were used for differential intron retention. Read counts were evaluated using multiple Fisher’s exact tests in three two-by-two tables using R, and *p*-values were adjusted using the Bayesian posterior probability method and considered significant at *p* < 0.05.

### In vivo mouse experiment

The tumor xenograft study was performed according to the protocol (#A22-29) approved by the Hiroshima University Research Facilities of Laboratory Animal Science. Female athymic nude mice (BALB/cAJcl-nu/nu, 5 to 6 weeks old) were purchased from CLEA Japan (Shizuoka, Japan). WT and LATS1/2 KO CAL27 cells were transplanted into both flanks (2 million cells per tumor) of each mouse. When the average tumor volume reached a predetermined value (~150 mm^3^) the mice were randomized into groups (10 tumors per group). Indisulam (Selleck Chemicals, 25 mg/kg/day) or the control diluent (3.5% DMSO, 6.5% Tween 80, 90% saline) was injected into the mice intraperitoneally. The mice were euthanized at the indicated time points (or when control-treated mice succumbed to the disease, as determined by the protocol).

### Statistical analysis

GraphPad Prism version 9 for Windows (GraphPad Software, CA) was used for statistical analysis. The data were analyzed by student’s *t*-test (two-sided) and analysis of variance (ANOVA) with Tukey–Kramer post hoc test. All experiments were repeated independently three times with similar results. All samples showed equal SE.

### Supplementary information

Supplementary information

Supplementary Table 1

## Data Availability

The DEPMAP data set is available online (https://depmap.org/portal/). All other data that supporting the findings of this study are available from the corresponding author upon reasonable request. Raw and processed RNA-seq data have been deposited in NCBI’s Gene Expression Omnibus (GEO) and can be accessed under the GEO Series Accession Number GSE (GSE268568).
