# Supplementary material for: YAP/TAZ interacts with RBM39 to confer resistance against indisulam
Source: Oncogenesis. 2024 Jul 15;13(1):25. doi: 10.1038/s41389-024-00527-0 (PMC11247092; doi:10.1038/s41389-024-00527-0)
Supplement: Supplementary file 1 — Supplementary information [file 41389_2024_527_MOESM1_ESM.pdf]

Supplementary Information

Supplementary Methods

DNA constructs

Plasmid pQCXIH-Myc-YAP was a gift from Kunliang Guan (Addgene plasmid # 33091 ; http://n2t.net/addgene:33091 ; RRID:Addgene_33091).

Immunofluorescence

Immunofluorescence was performed as described previously[4]. We used the primary antibody against YAP, RBM39, Alexa Fluor 594 and 488 secondary antibodies (Molecular Probes, Eugene, OR). DAPI (Molecular Probes, Eugene, OR) was applied for nuclear staining.

siRNA transfection

Cells were transfected with siRNA using the Lipofectamine RNAimax Reagent (Thermo fisher scientific). The siRNA sequences for RBM39 (#2:SASI_Hs02_0033-8551, #3:SASI_Hs01_00232189), and the negative control (MISSION® siRNA Universal Negative Control #2) were purchased from Sigma Aldrich. The siRNA for YAP (SMARTpool siGENOME YAP1, #M-012200-00-0005) was purchased from Dharmacon (CO).

Supplementary figures

a

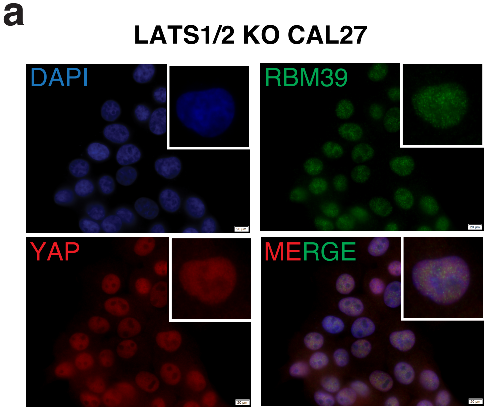

b

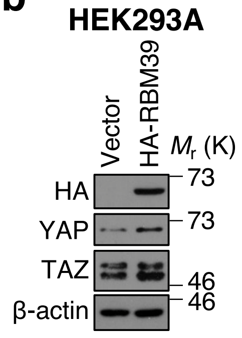

c

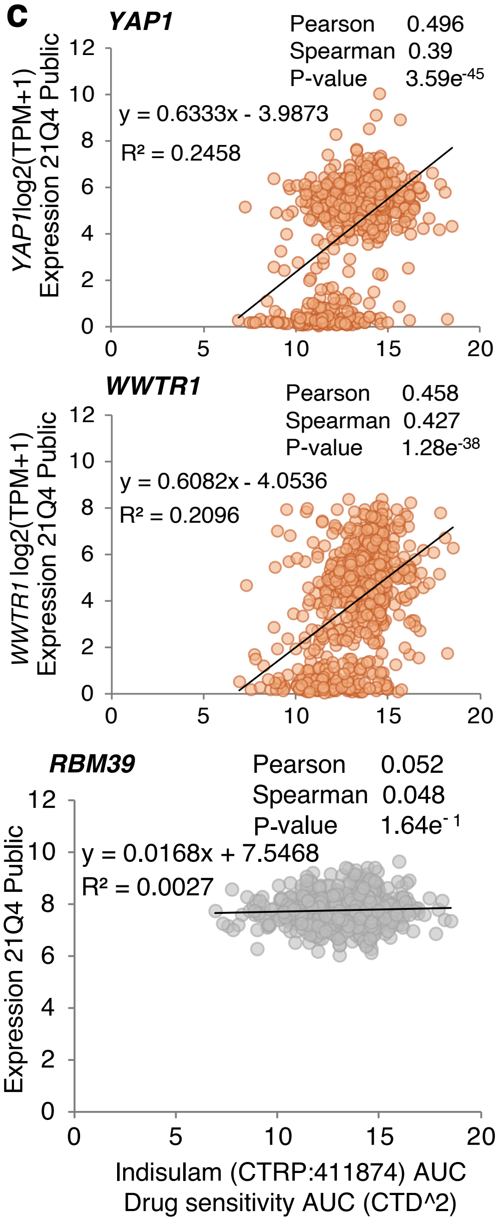

d

Top 20 gene expressions for indisulam sensitivity

Rank	Gene	Pearson	P-value
1	*WAS*	-0.473	1.14e-40
2	*AGAP2*	-0.467	9.75e-40
3	*SH2D3C*	-0.466	1.51e-39
4	*MYB*	-0.466	1.89e-39
5	*RASGRP2*	-0.463	6.30e-39
6	*IKZF1*	-0.463	6.62e-39
7	*NCKAP1L*	-0.454	2.25e-37
8	*ANKLE1*	-0.446	6.35e-36
9	*RASAL3*	-0.442	3.09e-35
10	*RHOH*	-0.438	2.77e-34
11	*CORO1A*	-0.438	1.42e-34
12	*PRKCB*	-0.437	2.07e-34
13	*PPP1R16B*	-0.436	2.96e-34
14	*PTPN7*	-0.436	3.13e-34
15	*SPN*	-0.44	5.97e-34
16	*TRAF3IP3*	-0.433	1.06e-33
17	*ARHGAP9*	-0.431	2.07e-33.
18	*SEPTIN6*	-0.43	2.91e-33.
19	*CD53*	-0.43	3.39e-33
20	*DCAF15*	-0.429	2.17e-33

e

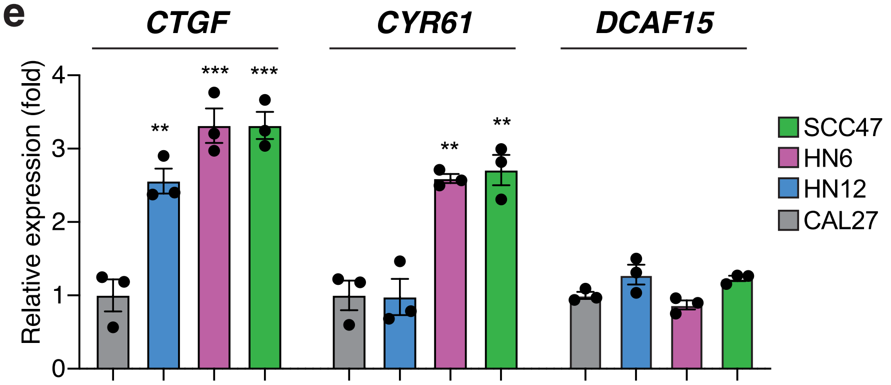

f

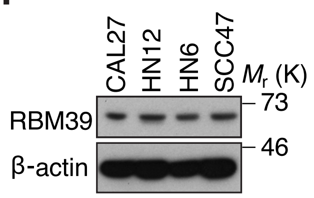

Supplementary figure 1

(**a**) Immunofluorescence of YAP and RBM39 in LATS1/2 KO CAL27 cells. (**b**) Western blot of HEK293A cells overexpressing vector or HA-RBM39. (**c**) Representative graph showing correlation between *YAP1*, *WWTR1*, *RBM39*, and indisulam sensitivity (AUC). (**d**) Top 20 gene expressions positively correlated with indisulam sensitivity. (**e**) Relative mRNA expression of *CTGF*, *CYR61*, *DCAF15* in HNSCC cell lines. (N=3) (**f**) Western blot of HNSCC cell lines. ANOVA with Tukey−Kramer post hoc test was used. Mean±SEM (**e**); ***, $P < 0.001$; **, $P < 0.01$ (compared to CAL27).

a

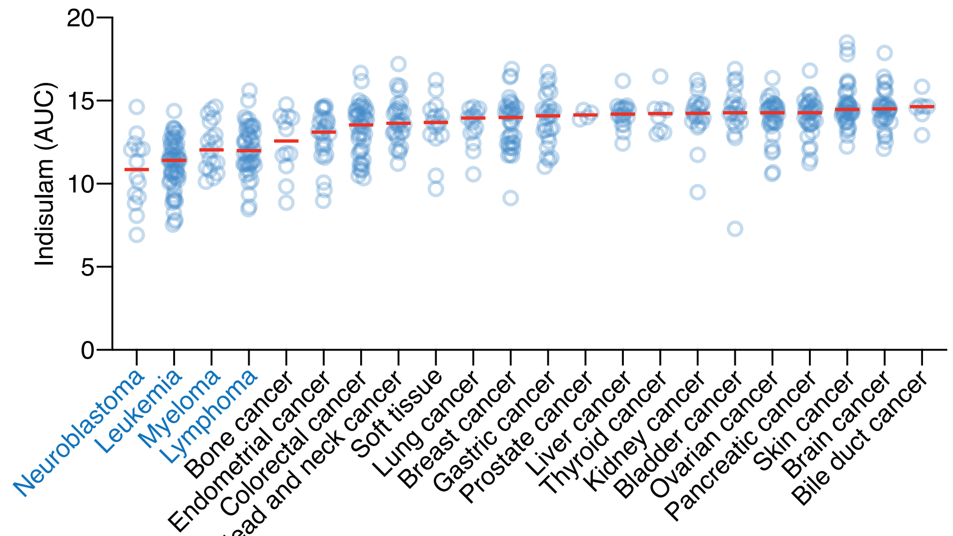

b

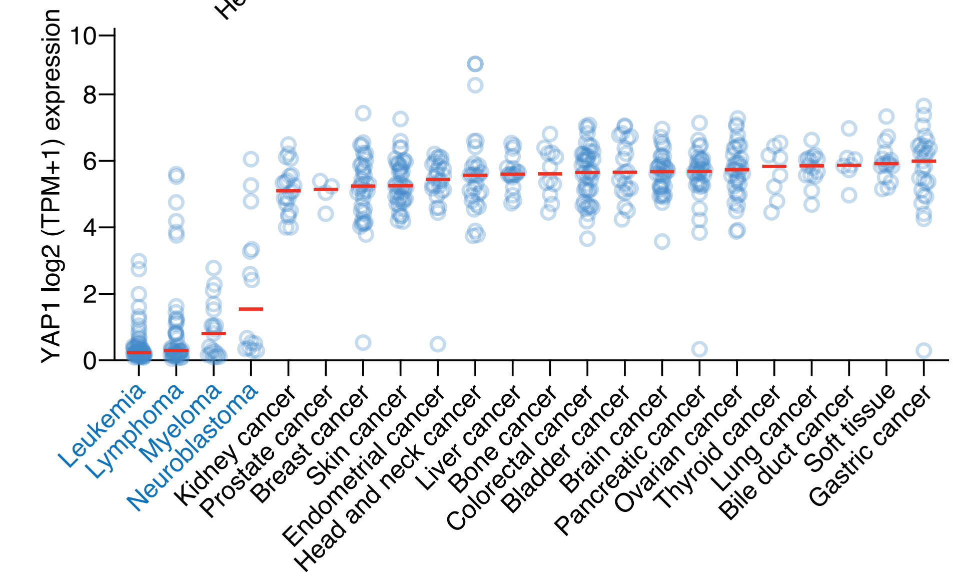

c

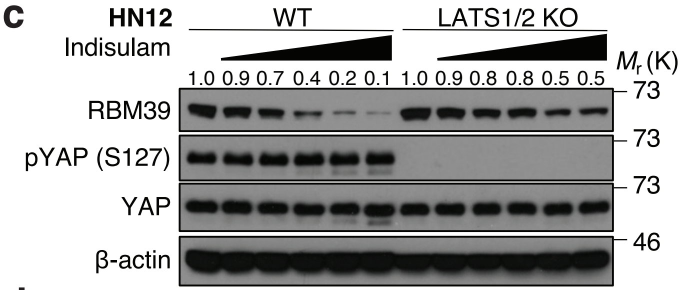

d

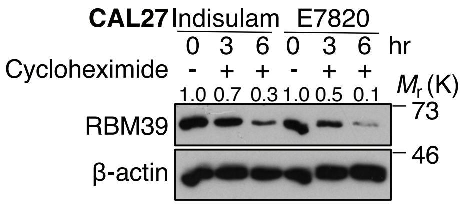

e

Gene name	Pearson	P-value
ITGA2	0.349	1.19e-21
ITGA3	0.485	4.47e-43
ITGB6	0.208	2.20e-8
COL4A4	0.077	4.10e-2
COL4A5	0.256	4.24e-12
COL4A6	0.200	7.93e-8
COL7A1	0.268	3.89e-13
COL16A1	0.258	3.32e-12
PTK2	0.253	8.17e-12

Supplementary figure 2

(**a**) Median indisulam area-under-curve (AUC) in cell lines from 26 tumor origins. Data was acquired from the CTD^2 network, each circle represents one cell line. (**b**) Median *YAP1* expression in the same data explained above. (**c**) Western blot of WT and LATS1/2 KO HN12 cells. Cells were treated with indisulam at 0, 0.01, 0.05, 0.1, 0.5, and 1 μM for 24 h. (**d**) Western blot of WT CAL27 cells. Cells were treated with cycloheximide (200 ng/μL) as well as indisulam (1 μM), and E7820 (1 μM) for 0, 3, 6 hr. (**e**) The DEPMAP shows the gene expressions conferring resistance to indisulam (Pearson ratio is gene expression vs drug sensitivity AUC). Protein level of RBM39 was compared to without Indisulam and cycloheximide treatment (**c** and **d**).

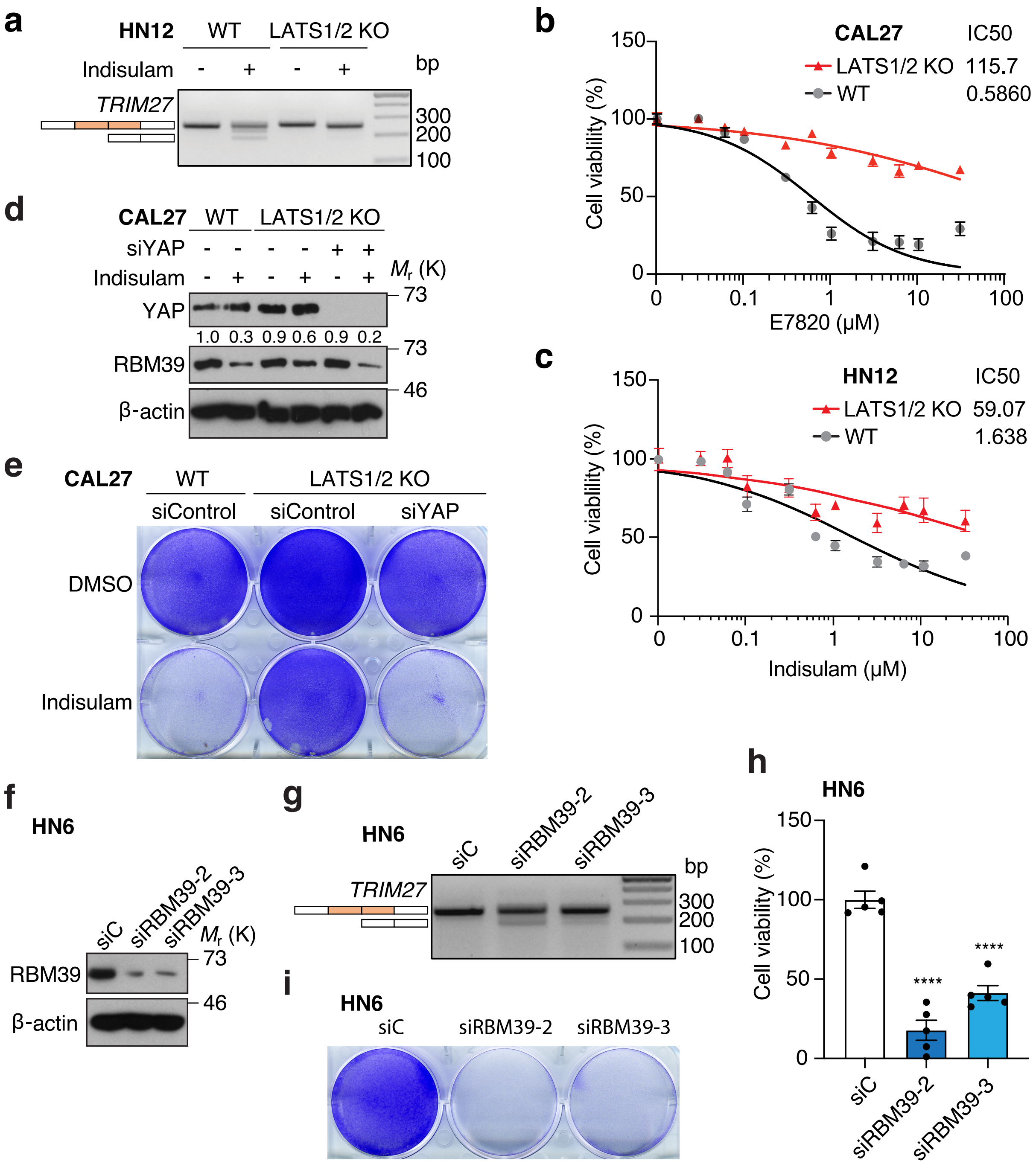

Supplementary figure 3

(**a**) Alternative splicing of *TRIM27* in WT and LATS1/2 KO HN12 cells treated with indisulam. (**b**) Cell viability of WT and LATS1/2 KO CAL27 cells treated with E7820. (N=3) (**c**) Cell viability of WT and LATS1/2 KO HN12cells treated with indisulam. (N=3) (**d**) Western blot of

CAL27 WT and LATS1/2 KO cells transfected with 30 nM of siRNA for control and YAP. Cells were treated with indisulam at 1 μM for 24 h. (**e**) Crystal violet staining. (**f**) Western blot of HN6 cells. Cells were transfected with siControl, siRBM39-2, or siRBM39-3. (**g**) Alternative splicing of *TRIM27* in HN6 cells transfected with siRNA. (**h**) Cell viability. (N=3) (**i**) Crystal violet staining. ANOVA with Tukey−Kramer post hoc test was used. Mean±SEM (**h**); ****, $P < 0.0001$ (compared to siC). Protein level of RBM39 was compared without siYAP and Indisulam treatment (**d**).
