# Supplementary material for: YAP/TAZ interacts with RBM39 to confer resistance against indisulam
Source: Oncogenesis. 2024 Jul 15;13(1):25. doi: 10.1038/s41389-024-00527-0 (PMC11247092; doi:10.1038/s41389-024-00527-0)
Supplement: Supplementary file 2 — Supplementary Table 1 [file 41389_2024_527_MOESM2_ESM.pdf]

# Supplementary Table 1

| Number | Uniprot Ac. | Gene Symbo | Speciecs | Identified Peptide | Total Unique Peptide | Protein Group Score | Reported in BioGRID |
|---|---|---|---|---|---|---|---|
| 1 | P15924 | DSP | Homo sapiens | 35 | 35 | 0.967029601 | + |
| 2 | Q15149 | PLEC | Homo sapiens | 31 | 30 | 0.965127699 | - |
| 3 | P13647 | KRT5 | Homo sapiens | 21 | 18 | 0.968679599 | - |
| 4 | P05787 | KRT8 | Homo sapiens | 20 | 17 | 0.9680098 | - |
| 5 | P02538 | KRT6A | Homo sapiens | 19 | 3 | 0.9636342 | + |
| 6 | P02533 | KRT14 | Homo sapiens | 18 | 5 | 0.964764699 | + |
| 7 | P48668 | KRT6C | Homo sapiens | 17 | 1 | 0.962508801 | + |
| 8 | Q13428 | TCOF1 | Homo sapiens | 13 | 13 | 0.966971699 | - |
| 9 | Q8IY63 | AMOTL1 | Homo sapiens | 13 | 12 | 0.9659006 | + |
| 10 | Q04695 | KRT17 | Homo sapiens | 12 | 7 | 0.968422499 | - |
| 11 | P08779 | KRT16 | Homo sapiens | 12 | 2 | 0.965186801 | + |
| 12 | Q9Y2J4 | AMOTL2 | Homo sapiens | 9 | 8 | 0.967262201 | + |
| 13 | P19012 | KRT15 | Homo sapiens | 9 | 2 | 0.9648479 | + |
| 14 | P04264 | KRT1 | Homo sapiens | 8 | 8 | 0.9682342 | + |
| 15 | Q14498 | RBM39 | Homo sapiens | 8 | 8 | 0.967891201 | + |
| 16 | P05783 | KRT18 | Homo sapiens | 8 | 7 | 0.967793401 | - |
| 17 | Q9BZE4 | GTPBP4 | Homo sapiens | 8 | 8 | 0.9655318 | - |
| 18 | P68371 | TUBB4B | Homo sapiens | 7 | 1 | 0.968153 | + |
| 19 | P46937 | YAP1 | Homo sapiens | 7 | 7 | 0.9678468 | |
| 20 | P07437 | TUBB | Homo sapiens | 7 | 1 | 0.967420202 | + |
| 21 | Q9H0A0 | NAT10 | Homo sapiens | 7 | 7 | 0.965985201 | - |
| 22 | Q15678 | PTPN14 | Homo sapiens | 7 | 7 | 0.965709001 | + |
| 23 | P11021 | HSPA5 | Homo sapiens | 7 | 6 | 0.962618601 | + |
| 24 | Q7KZI7 | MARK2 | Homo sapiens | 7 | 6 | 0.9612027 | - |
| 25 | P11142 | HSPA8 | Homo sapiens | 7 | 5 | 0.960448399 | + |
| 26 | Q8NI35 | PATJ | Homo sapiens | 7 | 7 | 0.959658399 | + |
| 27 | P13646 | KRT13 | Homo sapiens | 7 | 1 | 0.940876 | - |
| 28 | Q9BU76 | MMTAG2 | Homo sapiens | 6 | 6 | 0.9691069 | - |
| 29 | Q9Y383 | LUC7L2 | Homo sapiens | 6 | 4 | 0.9681485 | - |
| 30 | P0DMV8 | HSPA1A | Homo sapiens | 6 | 4 | 0.967459898 | + |
| 31 | Q3KQU3 | MAP7D1 | Homo sapiens | 6 | 6 | 0.9656781 | - |
| 32 | Q9NWB6 | ARGLU1 | Homo sapiens | 6 | 6 | 0.963084299 | - |
| 33 | O43290 | SART1 | Homo sapiens | 6 | 6 | 0.9625789 | - |
| 34 | Q5C9Z4 | NOM1 | Homo sapiens | 6 | 6 | 0.961941399 | - |
| 35 | Q9H0S4 | DDX47 | Homo sapiens | 5 | 5 | 0.968707699 | - |
| 36 | Q9BVP2 | GNL3 | Homo sapiens | 5 | 5 | 0.966972001 | - |
| 37 | P62701 | RPS4X | Homo sapiens | 5 | 5 | 0.9667955 | + |
| 38 | Q99959 | PKP2 | Homo sapiens | 5 | 5 | 0.966468699 | - |
| 39 | Q9UHD8 | SEPTIN9 | Homo sapiens | 5 | 5 | 0.9663064 | - |
| 40 | Q9BYG3 | NIFK | Homo sapiens | 5 | 5 | 0.9660125 | - |
| 41 | P08238 | HSP90AB1 | Homo sapiens | 5 | 3 | 0.965897098 | + |
| 42 | O15042 | U2SURP | Homo sapiens | 5 | 5 | 0.9657971 | - |
| 43 | Q5T0W9 | FAM83B | Homo sapiens | 5 | 5 | 0.965705801 | - |
| 44 | Q14244 | MAP7 | Homo sapiens | 5 | 5 | 0.965696499 | - |
| 45 | Q9NXE8 | CWC25 | Homo sapiens | 5 | 5 | 0.965631898 | - |
| 46 | P42766 | RPL35 | Homo sapiens | 5 | 5 | 0.965020001 | - |
| 47 | Q6ZRV2 | FAM83H | Homo sapiens | 5 | 5 | 0.963635702 | - |
| 48 | P08670 | VIM | Homo sapiens | 5 | 4 | 0.962415598 | + |
| 49 | Q16630 | CPSF6 | Homo sapiens | 4 | 4 | 0.968942801 | + |
| 50 | P46776 | RPL27A | Homo sapiens | 4 | 4 | 0.968085099 | + |

| | | | | | | | |
|---|---|---|---|---|---|---|---|
| 51 | P82914 | MRPS15 | Homo sapiens | 4 | 4 | 0.967847899 | - |
| 52 | Q9NP64 | ZCCHC17 | Homo sapiens | 4 | 4 | 0.9677274 | - |
| 53 | Q8TA86 | RP9 | Homo sapiens | 4 | 4 | 0.966806099 | - |
| 54 | Q9BRR8 | GPATCH1 | Homo sapiens | 4 | 4 | 0.966799501 | - |
| 55 | P18621 | RPL17 | Homo sapiens | 4 | 4 | 0.966510899 | + |
| 56 | Q9P031 | CCDC59 | Homo sapiens | 4 | 4 | 0.965932898 | - |
| 57 | Q07020 | RPL18 | Homo sapiens | 4 | 4 | 0.964865301 | + |
| 58 | Q9NY93 | DDX56 | Homo sapiens | 4 | 4 | 0.964811299 | - |
| 59 | P61254 | RPL26 | Homo sapiens | 4 | 4 | 0.963646401 | - |
| 60 | Q6SJ93 | FAM111B | Homo sapiens | 4 | 4 | 0.9636418 | - |
| 61 | P23396 | RPS3 | Homo sapiens | 4 | 4 | 0.963134199 | + |
| 62 | Q66PJ3 | ARL6IP4 | Homo sapiens | 4 | 4 | 0.962820798 | - |
| 63 | Q86SQ0 | PHLDB2 | Homo sapiens | 4 | 4 | 0.9626843 | - |
| 64 | P26373 | RPL13 | Homo sapiens | 4 | 4 | 0.962512702 | + |
| 65 | Q86X95 | CIR1 | Homo sapiens | 4 | 4 | 0.960855 | - |
| 66 | Q8N3R9 | MPP5 | Homo sapiens | 4 | 4 | 0.9608032 | + |
| 67 | P35240 | NF2 | Homo sapiens | 4 | 4 | 0.960519802 | + |
| 68 | P18124 | RPL7 | Homo sapiens | 4 | 4 | 0.958875801 | + |
| 69 | Q9UHR4 | BAIAP2L1 | Homo sapiens | 4 | 4 | 0.957478601 | - |
| 70 | Q9UGN5 | PARP2 | Homo sapiens | 4 | 4 | 0.954547498 | - |
| 71 | P13645 | KRT10 | Homo sapiens | 4 | 3 | 0.953812599 | - |
| 72 | P58107 | EPPK1 | Homo sapiens | 4 | 3 | 0.9522071 | - |
| 73 | P08727 | KRT19 | Homo sapiens | 4 | 1 | 0.943240799 | + |
| 74 | P62805 | H4C1 | Homo sapiens | 3 | 3 | 0.968265198 | - |
| 75 | P46779 | RPL28 | Homo sapiens | 3 | 3 | 0.968230098 | - |
| 76 | P62269 | RPS18 | Homo sapiens | 3 | 3 | 0.9674838 | + |
| 77 | Q8N5F7 | NKAP | Homo sapiens | 3 | 3 | 0.9674256 | - |
| 78 | P17066 | HSPA6 | Homo sapiens | 3 | 1 | 0.967099901 | + |
| 79 | P60709 | ACTB | Homo sapiens | 3 | 3 | 0.967064001 | + |
| 80 | Q8IY81 | FTSJ3 | Homo sapiens | 3 | 3 | 0.967039999 | - |
| 81 | P62263 | RPS14 | Homo sapiens | 3 | 3 | 0.966180999 | + |
| 82 | Q14134 | TRIM29 | Homo sapiens | 3 | 3 | 0.966173001 | - |
| 83 | Q9BUF5 | TUBB6 | Homo sapiens | 3 | 1 | 0.965848502 | + |
| 84 | P07900 | HSP90AA1 | Homo sapiens | 3 | 2 | 0.965656899 | + |
| 85 | Q9BZI7 | UPF3B | Homo sapiens | 3 | 3 | 0.9654193 | - |
| 86 | P62280 | RPS11 | Homo sapiens | 3 | 3 | 0.9653842 | - |
| 87 | P61247 | RPS3A | Homo sapiens | 3 | 3 | 0.965378001 | + |
| 88 | Q9UHK0 | NUFIP1 | Homo sapiens | 3 | 3 | 0.9651898 | - |
| 89 | P20042 | EIF2S2 | Homo sapiens | 3 | 3 | 0.9651521 | - |
| 90 | P35268 | RPL22 | Homo sapiens | 3 | 3 | 0.965025298 | - |
| 91 | P62081 | RPS7 | Homo sapiens | 3 | 3 | 0.964603402 | + |
| 92 | Q9NUL7 | DDX28 | Homo sapiens | 3 | 3 | 0.9642887 | - |
| 93 | O00622 | CCN1 | Homo sapiens | 3 | 3 | 0.963893302 | - |
| 94 | P07910 | HNRNPC | Homo sapiens | 3 | 3 | 0.9636181 | + |
| 95 | P10412 | H1-4 | Homo sapiens | 3 | 3 | 0.963158399 | - |
| 96 | P37108 | SRP14 | Homo sapiens | 3 | 3 | 0.962636299 | - |
| 97 | Q96I25 | RBM17 | Homo sapiens | 3 | 3 | 0.962582 | - |
| 98 | O00541 | PES1 | Homo sapiens | 3 | 3 | 0.962425198 | - |
| 99 | Q8N9Q2 | SREK1IP1 | Homo sapiens | 3 | 3 | 0.961909998 | - |
| 100 | P68363 | TUBA1B | Homo sapiens | 3 | 3 | 0.961873598 | + |

| | | | | | | | |
|---|---|---|---|---|---|---|---|
| 101 | P62277 | RPS13 | Homo sapiens | 3 | 3 | 0.9617646 | + |
| 102 | Q8WXX5 | DNAJC9 | Homo sapiens | 3 | 3 | 0.961474601 | - |
| 103 | P62249 | RPS16 | Homo sapiens | 3 | 3 | 0.960028298 | + |
| 104 | P09874 | PARP1 | Homo sapiens | 3 | 3 | 0.959913801 | + |
| 105 | Q5T8P6 | RBM26 | Homo sapiens | 3 | 3 | 0.9595282 | - |
| 106 | Q9BXS6 | NUSAP1 | Homo sapiens | 3 | 3 | 0.958841901 | - |
| 107 | Q9UMS4 | PRPF19 | Homo sapiens | 3 | 3 | 0.957348399 | - |
| 108 | Q7L014 | DDX46 | Homo sapiens | 3 | 3 | 0.957199901 | - |
| 109 | Q9UNQ2 | DIMT1 | Homo sapiens | 3 | 3 | 0.956909198 | - |
| 110 | P27448 | MARK3 | Homo sapiens | 3 | 2 | 0.956362199 | - |
| 111 | P46781 | RPS9 | Homo sapiens | 3 | 3 | 0.956073798 | + |
| 112 | P83731 | RPL24 | Homo sapiens | 3 | 3 | 0.953158502 | + |
| 113 | P00558 | PGK1 | Homo sapiens | 3 | 3 | 0.953043099 | + |
| 114 | Q9ULX6 | AKAP8L | Homo sapiens | 3 | 3 | 0.950425901 | - |
| 115 | Q9NQ29 | LUC7L | Homo sapiens | 3 | 1 | 0.950123899 | - |
| 116 | Q9Y3D9 | MRPS23 | Homo sapiens | 3 | 3 | 0.949513599 | - |
| 117 | O15027 | SEC16A | Homo sapiens | 3 | 3 | 0.946510799 | + |
| 118 | Q9UQ35 | SRRM2 | Homo sapiens | 3 | 3 | 0.935774803 | + |
| 119 | Q5JTH9 | RRP12 | Homo sapiens | 2 | 2 | 0.967562102 | - |
| 120 | P56182 | RRP1 | Homo sapiens | 2 | 2 | 0.967528202 | + |
| 121 | O00488 | ZNF593 | Homo sapiens | 2 | 2 | 0.967274498 | - |
| 122 | P27635 | RPL10 | Homo sapiens | 2 | 2 | 0.967211202 | + |
| 123 | P62851 | RPS25 | Homo sapiens | 2 | 2 | 0.9667992 | + |
| 124 | P62424 | RPL7A | Homo sapiens | 2 | 2 | 0.966703299 | + |
| 125 | Q9ULE0 | WWC3 | Homo sapiens | 2 | 2 | 0.9664183 | + |
| 126 | P62910 | RPL32 | Homo sapiens | 2 | 2 | 0.965552501 | - |
| 127 | O95232 | LUC7L3 | Homo sapiens | 2 | 2 | 0.9654583 | - |
| 128 | P11387 | TOP1 | Homo sapiens | 2 | 2 | 0.965266202 | - |
| 129 | P21796 | VDAC1 | Homo sapiens | 2 | 2 | 0.964786701 | - |
| 130 | P62861 | FAU | Homo sapiens | 2 | 2 | 0.964567501 | - |
| 131 | Q9H8W3 | FAM204A | Homo sapiens | 2 | 2 | 0.9645143 | - |
| 132 | Q9NUP9 | LIN7C | Homo sapiens | 2 | 2 | 0.964417301 | + |
| 133 | Q6KB66 | KRT80 | Homo sapiens | 2 | 1 | 0.964110401 | - |
| 134 | Q9UMY1 | NOL7 | Homo sapiens | 2 | 2 | 0.963659 | - |
| 135 | P62899 | RPL31 | Homo sapiens | 2 | 2 | 0.963408198 | + |
| 136 | P40429 | RPL13A | Homo sapiens | 2 | 2 | 0.963297699 | + |
| 137 | P07384 | CAPN1 | Homo sapiens | 2 | 2 | 0.9622223 | + |
| 138 | Q13427 | PPIG | Homo sapiens | 2 | 2 | 0.961244199 | - |
| 139 | Q9UJV9 | DDX41 | Homo sapiens | 2 | 2 | 0.961127602 | - |
| 140 | Q9Y446 | PKP3 | Homo sapiens | 2 | 2 | 0.9608678 | - |
| 141 | P0CG47 | UBB | Homo sapiens | 2 | 2 | 0.960339699 | - |
| 142 | P16401 | H1-5 | Homo sapiens | 2 | 2 | 0.9601711 | - |
| 143 | P14625 | HSP90B1 | Homo sapiens | 2 | 1 | 0.959571999 | + |
| 144 | P83881 | RPL36A | Homo sapiens | 2 | 2 | 0.959396999 | - |
| 145 | P68104 | EEF1A1 | Homo sapiens | 2 | 2 | 0.959196702 | + |
| 146 | Q96AG4 | LRRC59 | Homo sapiens | 2 | 2 | 0.959168501 | + |
| 147 | Q99459 | CDC5L | Homo sapiens | 2 | 2 | 0.958721802 | - |
| 148 | O60814 | H2BC12 | Homo sapiens | 2 | 1 | 0.958711401 | - |
| 149 | P38646 | HSPA9 | Homo sapiens | 2 | 2 | 0.958102599 | + |
| 150 | Q9UHR5 | SAP30BP | Homo sapiens | 2 | 2 | 0.957643799 | - |

| | | | | | | | |
|---|---|---|---|---|---|---|---|
| 151 | P62266 | RPS23 | Homo sapiens | 2 | 2 | 0.9570699 | + |
| 152 | P62937 | PPIA | Homo sapiens | 2 | 2 | 0.9563636 | + |
| 153 | Q8NHQ9 | DDX55 | Homo sapiens | 2 | 2 | 0.9562742 | - |
| 154 | Q9H5V9 | CXorf56 | Homo sapiens | 2 | 2 | 0.954596698 | - |
| 155 | Q4KMP7 | TBC1D10B | Homo sapiens | 2 | 2 | 0.9542857 | - |
| 156 | P06899 | H2BC11 | Homo sapiens | 2 | 1 | 0.954081301 | - |
| 157 | P00338 | LDHA | Homo sapiens | 2 | 2 | 0.953873601 | + |
| 158 | P07305 | H1-0 | Homo sapiens | 2 | 2 | 0.9537335 | - |
| 159 | Q8WVM0 | TFB1M | Homo sapiens | 2 | 2 | 0.951755099 | - |
| 160 | Q02297 | NRG1 | Homo sapiens | 2 | 2 | 0.9514778 | - |
| 161 | Q9H4L5 | OSBPL3 | Homo sapiens | 2 | 2 | 0.948822901 | - |
| 162 | Q92522 | H1-10 | Homo sapiens | 2 | 2 | 0.948582999 | - |
| 163 | P62913 | RPL11 | Homo sapiens | 2 | 2 | 0.948316898 | + |
| 164 | Q96EU6 | RRP36 | Homo sapiens | 2 | 2 | 0.947031301 | + |
| 165 | P13796 | LCP1 | Homo sapiens | 2 | 2 | 0.944670901 | - |
| 166 | P49761 | CLK3 | Homo sapiens | 2 | 2 | 0.942912798 | - |
| 167 | P05114 | HMGN1 | Homo sapiens | 2 | 2 | 0.940173201 | - |
| 168 | P08133 | ANXA6 | Homo sapiens | 2 | 2 | 0.939398699 | - |
| 169 | P13010 | XRCC5 | Homo sapiens | 2 | 2 | 0.939135998 | + |
| 170 | Q9H7H0 | METTL17 | Homo sapiens | 2 | 2 | 0.938306201 | - |
| 171 | Q9Y5J1 | UTP18 | Homo sapiens | 2 | 2 | 0.936112098 | - |
| 172 | P42166 | TMPO | Homo sapiens | 2 | 2 | 0.935194403 | + |
